# Genomic Integration and Germline Transmission of Plasmid Injected into Crustacean *Daphnia magna* Eggs

**DOI:** 10.1371/journal.pone.0045318

**Published:** 2012-09-18

**Authors:** Yasuhiko Kato, Tomoaki Matsuura, Hajime Watanabe

**Affiliations:** Department of Biotechnology, Graduate School of Engineering, Osaka University, Osaka, Japan; Oxford Brookes University, United Kingdom

## Abstract

The water flea, *Daphnia*, has been the subject of study in ecology, evolution, and environmental sciences for decades. Over the last few years, expressed sequence tags and a genome sequence have been determined. In addition, functional approaches of overexpression and gene silencing based on microinjection of RNAs into eggs have been established. However, the transient nature of these approaches prevents us from analyzing gene functions in later stages of development. To overcome this limitation, transgenesis would become a key tool. Here we report establishment of a transgenic line using microinjection of plasmid into *Daphnia magna* eggs. The green fluorescent protein (*GFP*) gene fused with the *D. magna* histone *H2B* gene under the control of a promoter/enhancer region of the *elongation factor 1α-1* (*EF1α-1*) gene, *EF1α-1::H2B-GFP*, was used as a reporter providing high resolution visualization of active chromatin. Transgenic lines were obtained from 0.67% of the total fertile adults that survived the injections. One of the transgenic animals, which exhibited fluorescence in the nuclei of cells during embryogenesis and oogenesis, had two copies of *EF1α-1::H2B-GFP* in a head-to-tail array. This is the first report of a transgenesis technique in *Daphnia* and, together with emerging genome sequences, will be useful for advancing knowledge of the molecular biology of *Daphnia*.

## Introduction

The water flea *Daphnia,* is a small crustacean that commonly inhabits fresh water ponds and lakes on all continents of the globe and has long been used as a model for understanding animal responses and adaptations to environmental changes [1]. It is also an attractive model species for understanding the evolution of reproductive strategies because it usually alternates between parthenogenetic and sexual reproduction as a consequence of environmental quality [2]. Their significance in ecology, evolutionary biology, and toxicology has prompted us to conduct genetic research on *Daphnia*. In 2005, expressed sequence tag (EST) sequences of *D. magna* were analyzed [3] and in 2011, the genome of a related daphniid, *D. pulex,* was described [4]. Using these sequence data, DNA microarrays have been developed for both *D. magna* [5] and *D. pulex* [6]. Thus, the combination of emerging genomic sequences with observations accumulated over decades make *Daphnia* a prime model of ecological and toxicological genomic research [4], [6], [7].

To understand the functions of newly discovered genes, reverse genetic tools are necessary. Recently, microinjection of double-stranded RNAs and capped polyadenylated RNAs into parthenogenetic eggs enabled us to perform loss- and gain-of-function analyses in embryos [8], [9]. However, the transient nature of these functional approaches makes it difficult to investigate gene functions in the adult stage and throughout the lifespan. Transgenesis may provide not only a tool to overcome these imitations, but also an excellent to monitor the developmental process *in vivo*. However, to date, no germline transmission has been reported for any *Daphnia* species. Here we report the generation of a transgenic line of *D. magna* expressing green fluorescent protein (GFP) fused with histone H2B under the control of the *elongation factor 1α-1* (*EF1α-1*) promoter/enhancer. Transgenic technology will allow functional analysis of target genes and of the development of specific cell populations in the crustacean *D. magna*.

## Materials and Methods

### Daphnia Strain and Culture Conditions

The *D. magna* strain (NIES clone) was obtained from the National Institute for Environmental Studies (NIES; Tsukuba, Japan) [10]. The strain originated at the Environmental Protection Agency (USA) and was maintained for more than 10 years at NIES. M4 culture medium [11] was prepared using charcoal-filtered tap water maintained at room temperature overnight prior to use. Cultures of 20 individuals per liter were incubated at 24±1°C under a 14 h light/10 h dark photoperiod. A 0.01 mL suspension of 4.3×10^8^ cells/mL *Chlorella* was added daily to each culture. Water hardness was between 72 and 83 mg/L CaCO_3_, pH was between 7.0 and 7.5, and dissolved oxygen concentration was between 80 and 99% of saturation.

### Genomic DNA Extraction

A standard phenol/chloroform method for mouse tail DNA extraction was modified and used for this study. The 10 adult female daphnids were homogenized using Micro Smash MS-100 (TOMY, Tokyo, Japan) at 3,000 rpm for 1.5 min in 765 µl of lysis buffer (1.18% SDS, 59 mM Tris-HCl, 23.6 mM EDTA, 1118 mM NaCl, pH7.5) and then 135 µl of Proteinase K solution (10 mg/ml, Nakalai tesque, Kyoto, Japan) was added to the tube. After incubation at 50°C overnight, the homogenate was extracted with phenol and phenol/chloroform/isoamyl alcohol (25:24:1). Then, 2 µl of RNaseA solution (20 mg/ml, Nakalai tesque) were added. After incubation at 37°C for 30 min, the DNA solution was further purified with phenol/chloroform/isoamyl alcohol (25:24:1) and chloroform. To precipitate DNA, equal volume of isopropanol was added, and the tube was centrifuged. The pellet was washed with 70% ethanol, briefly dried, and dissolved in TE buffer.

### Cloning of the EF1α-1 Promoter/enhancer and 3′UTR

A 2.7 kb genomic fragment including upstream sequences of the *EF1α-1* gene was from *D. magna* genomic DNA and cloned into pCR-Blunt-II TOPO (Invitrogen, Carlsbad, CA, USA). Primers to amplify the genomic fragment were (5'-3'): forward: ACCCAGAGGTTTCGGCTACATTGAAG and reverse: GTGGTCGACTTGCCAGAGTCTACGTG. From the 2.7 kb fragment, a 2.3 kb genomic fragment including upstream sequences, the transcription start site, the complete intron, and 43 bp of the second exon with a start Met codon was re-amplified and cloned into the HindIII/SmaI site of pRCS21 encodes the *DsRed2* gene [12], resulting in construction of pRCS21-EF1*α*1. In addition, a 522 bp of *EF1α-1* 3′ UTR fragment was amplified from *D. magna* cDNA, as prepared previously [13], and cloned into the NotI/EcoT22I site of pRCS-EF1*α*1 to generate pRCS21-EF1*α*1(UTR). Primers to amplify the cDNA fragment were (5'-3'): forward: GCGGCCGCATGGAGGCTACTATTCCATCCAACC and reverse: ATGCATGTCCAAATTATCTTGTATTGGAGC.

### Generation of Plasmids for Transgenesis

The coding sequence for the *D. magna* histone *H2B* gene was amplified from cDNA that was synthesized previously [13]. The resulting product was cloned into pCR2.1 TOPO (Invitrogen). The H2B fragment was cloned into the 5′-side of the DsRed2 coding sequence in pRCS21. Then, the DsRed2 coding sequence was replaced with the EGFP coding sequence to generate pCS-H2B-GFP. The resulting fusion was then re-amplified by PCR and cloned into the SmaI/NotI site of pRCS-EF1*α*1(UTR) to generate pCS-EF1*α*1::H2B-GFP (oligonucleotide sequences are available upon request).

### Transgenesis

Sixty-two point five ng/µl of pCS-EF1*α*1::H2B-GFP was injected into *D. magna* eggs just after ovulation according to established procedures [8]. GFP expression was detected with a Leica M165C fluorescence stereoscopic microscope (Leica Microsystems Heidelberg GmbH, Mannheim, Germany) equipped with a 480-nm excitation filter and a 510-nm barrier filter (filter set GFP2). Fluorescent images were recorded with a color digital camera (Leica DC500) mounted on the microscope.

### Southern Blot Analysis

Approximately 10 µg of genomic DNA were digested with BglII, PstI, HindII, and EcoRI, size-separated by agarose gel electrophoresis, and blotted onto positively charged nylon membranes (Hybond-N+; GE Healthcare, Little Chalfont, England). RNA probes were prepared with a DIG RNA labeling kit (Roche Diagnostic GmbH, Manheim, Germany). Primers to amplify templates for probe preparation were (5'-3'): forward: ATGGTGAGCAAGGGCGAGGA and reverse: TAATACGACTCACTATAGGGCCGCTACTTGTACAGCTC. The membrane was hybridized with DIG-labeled RNA probes for 16 hr at 50°C with DIG easy hyb (Roche Diagnostic). DIG-labeled RNA was detected with an alkaline phosphatase-conjugated anti-DIG antibody using CDP star (Roche Diagnostic) according to the manufacturer's protocol.

### Inverse PCR

Preparation of template for inverse PCR was performed using a standard protocol for *Drosophila* [14]. Approximately 2 µg of genome DNAs from HG-1 or HG2 transgenic line were digested with EcoRI or BamHI, and circularized by ligation for 16.5 h at 4°C. Primers to amplify a plasmid-to-genomic DNA junction were: HG-1 line, Forward (F) 5′-GCCGACCACTACCAGCAGAA-3′ and Reverse (R) 5′-GCTTGTCGGCCATGATATAGAC-3′; HG-2 line, F 5′-GGGGTTAATAAACTGGTGGTTACTGG-3′ and R 5′-ACACCCCGGTAAATAAGTGGAG-3′; PCR fragments were separated by electrophoresis in a 0.7% agarose gel, purified, cloned into a pBluescript vector using an In-fusion HD cloning kit (Takara Bio Inc., Shiga, Japan), and sequenced.

## Results and Discussion

### Generation of the Reporter Construct

**Figure 1:**
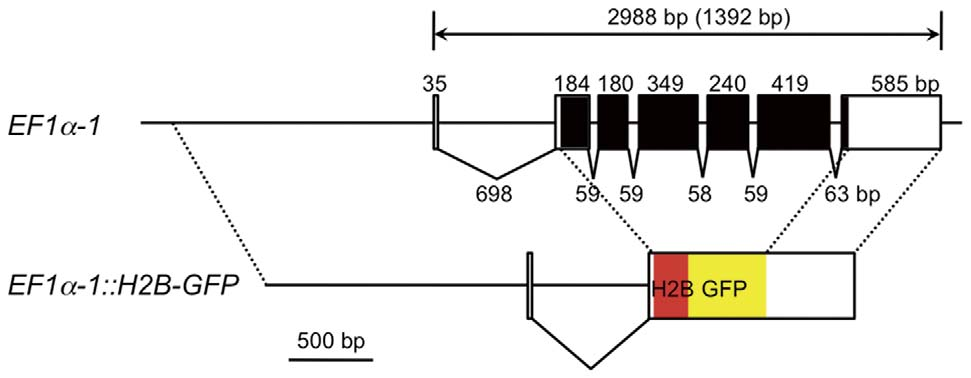
Structure of the *EF1α-1::H2B-GFP* construct. The upper diagram shows the genome structure of the *EF1α-1* gene. Untranslated regions and the coding region are indicated as white and black boxes, respectively. Sizes of introns and exons and total gene size are shown. The size of the coding region is also shown in parentheses. Annotated sequence data of the *EF1α-1* gene f have been deposited with the DDBJ/EMBL/Genbank Data Libraries under Accession No. AB734039. The lower diagram shows the structure of the *EF1α-1::H2B-GFP* gene. The H2B and GFP coding regions are shown as red and yellow boxes. Dashed lines indicate regions originated from the *EF1α-1* gene.

In this study we have used a histone H2B-EGFP fusion protein as a marker for transgenesis and its nuclear localization facilitates the visualization of individual cells [15]. To express the marker gene, we selected a promoter/enhancer of the *elongation factor 1α* (*EF1α*) gene, which drives ubiquitous expression from invertebrates to vertebrates. Analysis of the genome database revealed that *D. magna* has two *EF1α* genes, *EF1α-1* and *EF1α-2*. Only *EF1α-1* contains an intron within its 5′ untranslated region (Figure 1) that is evolutionarily conserved [16] and has an important role in strong expression [17], [18]. Consistent with this conserved genomic structure, expression of the *EF1α-1* gene was much higher than that of *EF1α-2* (data not shown), prompting us to use the *EF1α-1* promoter for maker gene expression. A 2.3 kb genomic fragment including upstream sequences, the transcription start site, the complete first intron, and part of the second exon with a start Met codon was fused in frame to an *H2B-GFP* gene. In addition, to mimic endogenous *EF1α-1* gene expression and proper processing of the 3' end of reporter mRNA, the full length of *EF1α-1* 3′UTR was added downstream of the *H2B-GFP* gene (Figure 1).

### Transgenesis and Expression of Marker Genes in G_0_ Embryos and Adults

**Figure 2:**
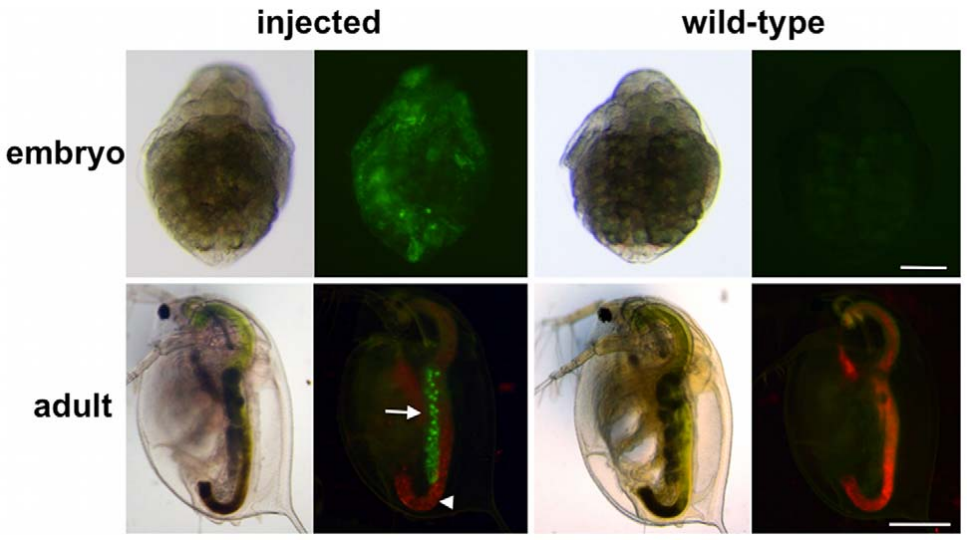
H2B-GFP expression in G_0_ transgenic animals. Upper and lower columns show embryos 27 h and adults 7 days after ovulation. Images were taken from the same sample under a bright-field microscope (left image) and a fluorescence microscope (right image). Left and right columns represent injected and wild-type animals, respectively. Wild-type animals show weak green autofluorescence. Red fluorescence, mainly detected in the gut that extended the body from anterior to posterior, is autofluorescence of chlorella, which is feed for daphnids. Arrows and arrowheads indicate ovaries and guts. Scale bar: 100 µm in the upper column; 500 µm in the lower column.

We injected the reporter plasmid into 562 eggs and observed GFP fluorescence during development. Mosaic nuclear localized fluorescence of *H2B-GFP* could be detected about 6 h after injection and was maintained in all 385 hatched embryos. During juvenile instar stages, *H2B-GFP* expressions in somatic tissues disappeared (Figure 2). These transient expressions possibly were from nonintegrated plasmid DNA. However, among the 298 surviving adults, two showed uniform H2B-GFP expression in the ovaries (Table 1, Figure 2); suggesting that germline transmission of injected DNAs occurred.

**Table 1:** Summary of the transgenesis experiment.

	Total number
Injected eggs	562
Hatched eggs	385
Juveniles	349
Adults	298
Germline transmission	2

### Germline Transmission and Maintenance of the Transgenic Line

**Figure 3:**
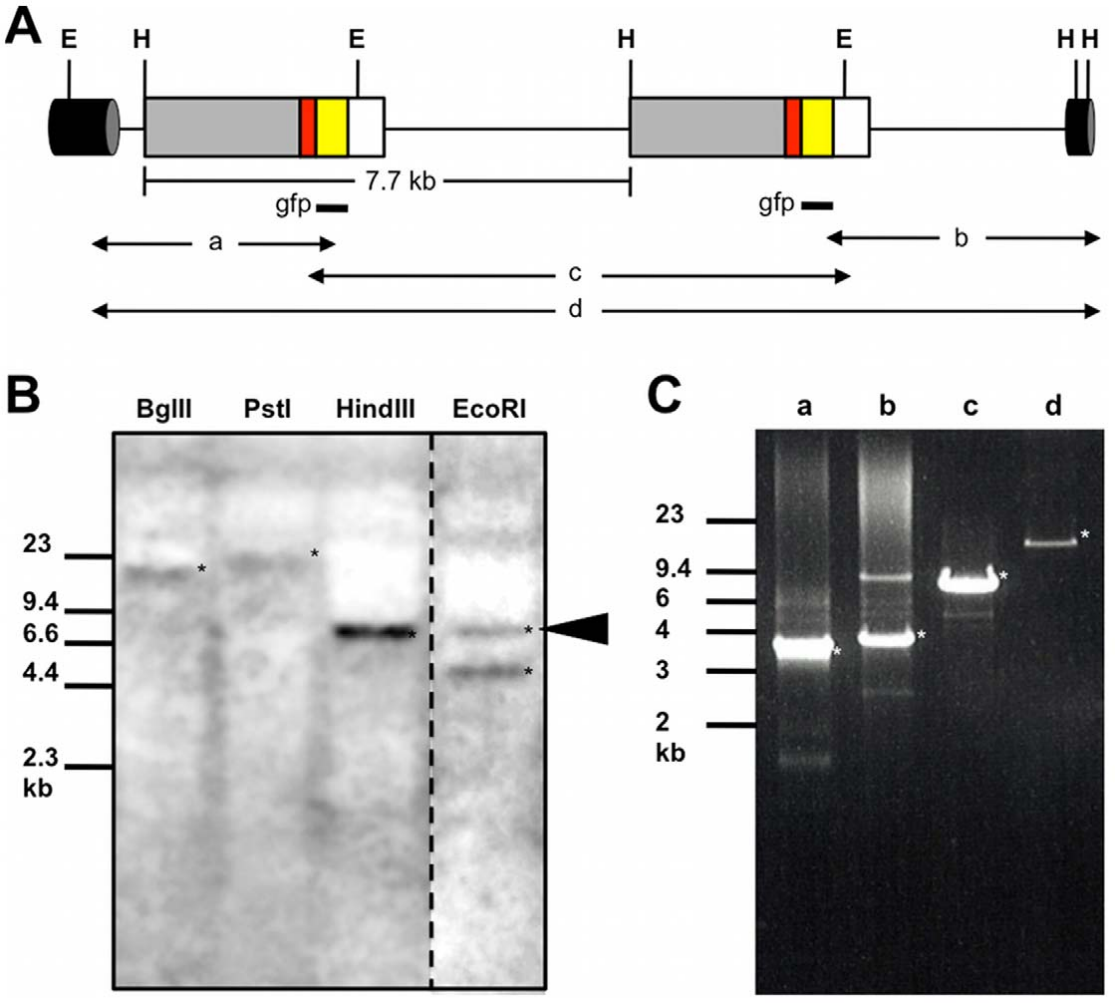
Characterization of the transgene structure. (A) Simplified diagram of the integrated transgene. Two copies are shown in a head-to-tail array. Grey, red, yellow, and white boxes indicate the EF-lα-1 promoter–enhancer region, H2B coding region, GFP coding region, and EF-lα-1 3′UTR, respectively. A cylinder shows chromosome DNA surrounding the transgene. Regions to be hybridized by the gfp probe were indicated as black bars. a, b, c, and d, indicate fragments amplified by PCR to confirm the structure of the transgene (see Figure 3C). EcoRI (E) and HindIII (H) sites outside the transgene were found in sequences of genomic contig42228 and scaffold466. (B) Southern blots of DNA hybridized with the gfp probe. An arrowhead indicates a 7.7 kb band whose length is the same as that of plasmid DNA. (C) PCR to confirm the transgene structure. Amplified genomic DNAs were resolved by agarose gel electrophoresis. Asterisks indicate DNA fragments from the integrated transgene. Migration of markers with lengths indicated (kb) is shown at the left.

To confirm integration of *EF1α-1::H2B-GFP* into the genome, transgene behavior was examined in one of the potential transgenic lines, HG-1. We first performed southern blots. Genomic DNA was digested with BglII or PstI, both of which do not cut the injected plasmid DNA sequence, and probed with the GFP coding sequence, resulting in a longer band than the full-length of the plasmid, 7.7 kb. In addition, genome digestion with EcoRI or HindIII, enzymes that cut single sites in the plasmid, produced a 7.7 kb band, suggesting insertion of the entire plasmid in tandem arrays (Figure 3A, 3B). Second, to identify the insertion site, we performed inverse PCR and cloned plasmid-to-genomic DNA junction fragments. Insertion was mapped to genomic contig42228 provided on early release data of the *D. magna* genome sequence. We also found a sequence from the scaffold466 upstream of the integration site, but this genomic sequence data was not enough to find any genes located near the transgenes. Finally, PCR reactions to amplify the partial and full length of the transgene revealed that two *EF1α-1::H2B-GFP* genes were tandemly integrated into the genome (Figure 3C).

### Monitoring Embryogenesis and Oogenesis Using the Transgenic Line

**Figure 4:**
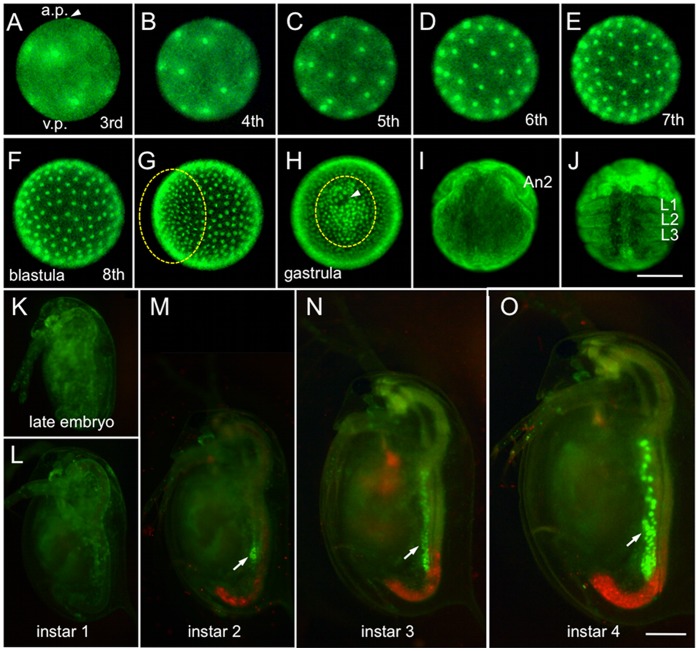
Visualization of embryogenesis and oogenesis. (A–F) The third to eighth cleavage stage for blastula formation. The egg has polarity. The upper side is the animal pole (a.p.); the lower side is the vegetal pole (v.p.). An arrowhead indicates the polar body. A blastula was formed 3 h 20 min after ovulation. (G) Early gastrulation (4 h 40 min after ovulation). (H) Late gastrulation (5 h 40 min after ovulation). Ventral views and anterior upwards. An arrowhead indicates a pit produced by invagination of the genital rudiment. Cells that migrate to the region to be the ventral side are circled by a dotted line. (I) Cephalic appendage developing stage (11 h 40 min after ovulation). (J) Early thoracic appendage developing stage (16 h after ovulation). Ventral view and anterior upwards. An2 second antenna, L1–L3 first to third thoracic segments. (K–O) Development between late embryonic and fourth instar stage. Lateral views with ventral to the left and anterior upwards. Arrows indicate fluorescence of H2B-GFP in ovaries. Red fluorescence is autofluorescence of chlorella. Scale bar: 100 µm in A–J; 250 µm in K–O.

*Daphnia* parthenogenetic eggs have been known to exhibit superficial cleavage [19], [20]. We followed early embryogenesis in the transgenic line HG-1 for 16 hour after ovulation (Figure 4). We could not clearly observe GFP fluorescence in the nucleus of embryos until the second cleavage stage, probably because the higher fluorescence of maternally supplied H2B-GFP protein in the cytosol prevented us from detecting the H2B-GFP protein (data not shown). The first 8 cleavages to produce a blastula took place every 20 minutes (Figure 4A–4F), except in some cells located at the vegetable pole, which may be presumptive germ cells (data not shown). After the 8th division, cell cycles became desynchronized, indicating that midblastula transition occurred at this stage. During gastrulation, cell migration to form the germ layer and invagination of a sheet of cells could be detected (Figure 4G, 4H). At the later stages, appendage formation was clearly observed (Figure 4I, 4J). These observations were consistent with those reported in previous studies with other cladocerans [21], [22].

**Figure 5:**
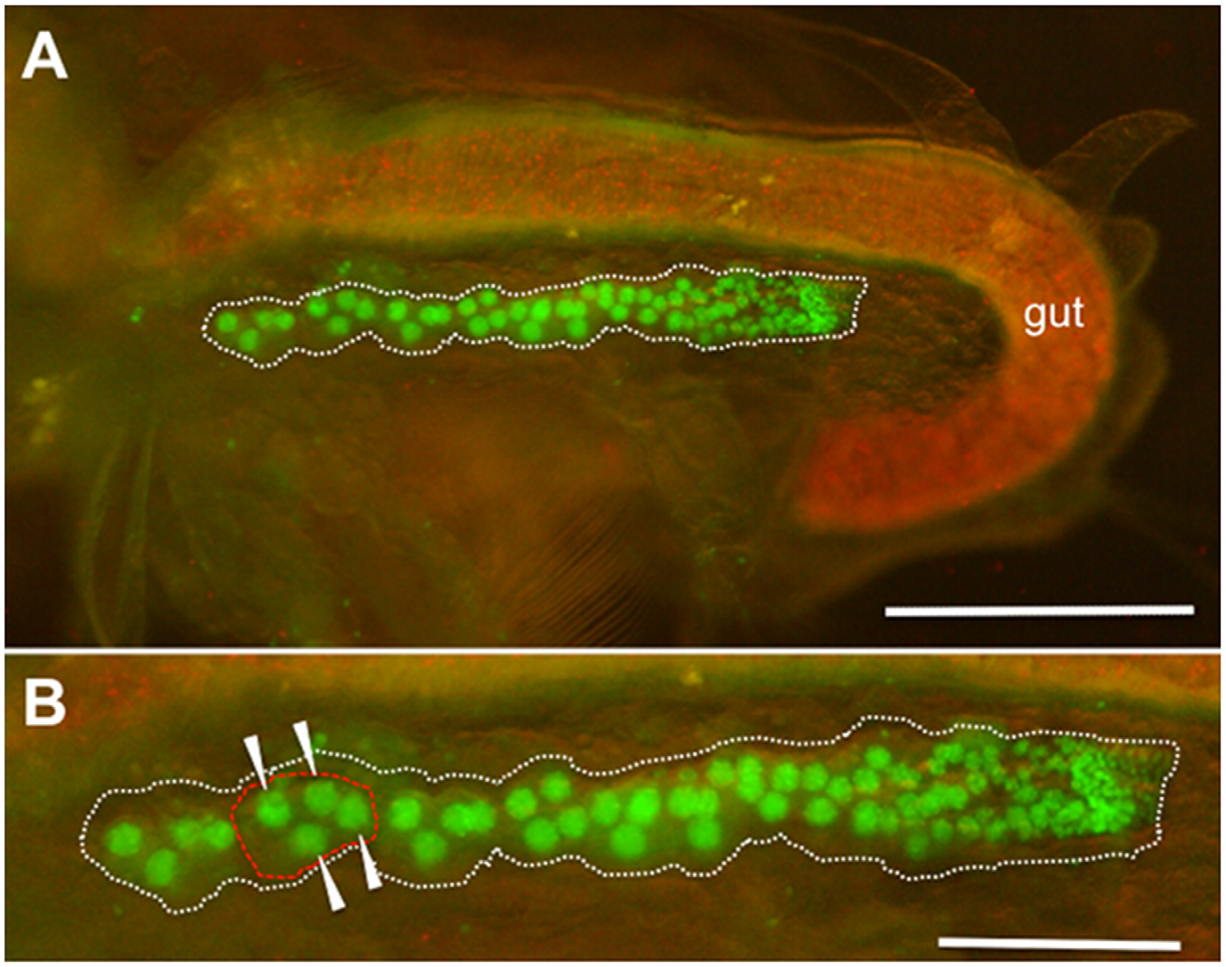
H2B-GFP expression in adult ovaries. Adult *D. magna* HG-1 line 2 h after ovulation was incubated in 50% ethanol for 1 min to stop movement and removed the carapace that covered the body. Lateral views with anterior to the left and dorsal upwards. (A) Fluorescence of a thorax and an abdomen in the adult HG-1 line. To show body structure, visible light illumination was also used. Red fluorescence in the gut shows autofluorescence of chlorella. (B) Magnified view of the ovary expressing H2B-GFP. Dotted white and red lines show an ovary and an oocyte cluster comprised of one oocyte and three nurse cells, which are indicated with arrowheads. Smaller nuclei are scattered throughout germarium located at the posterior end of the ovary.

After embryogenesis, uniform fluorescence of H2B-GFP throughout the body weakened (Figure 4K–4M). In contrast, at the second juvenile instar, ovaries started to exhibit strong fluorescence in the nuclei of germ cells (Figure 4M–4O). We could detect the germarium in the posterior end of the ovary and the 4-cell cystoblast that matures into an oocyte cluster of three nurses and the presumptive oocyte in the anterior region (Figure 5).

Another transgenic line, HG-2, was expressed not only in the ovary, but also in the other organs including second antennae, thoracic appendages. Inverse PCR analysis revealed that plasmid DNAs are inserted near the *disconnected* (*Disco*) gene located in the scaffold687 (data not shown). Differences of transgene behavior between HG-1 and HG-2 lines suggested that transgenic line-specific expression patterns of the H2B-GFP gene may be ascribed to position effects in the genomic integration site. The HG-1 and HG-2 transgenic lines have been maintained in our laboratory for more than 30 generations and 12 generations, respectively.

### Future Prospects

Here we report the first transgenic line in the cladoceran crustacean *Daphnia magna*. Transgenic lines can be obtained by direct introduction of plasmid DNA, which is the simplest method for transgenesis, and maintained stably for generations. Genomic integration of the exogenous gene, *EF1α-1::H2B-GFP*, enabled us to observe the dynamics of specific cell populations even in adults, demonstrating that transgenesis will be a useful tool to analyze gene functions throughout the lifespan of *D. magna*. However, the germline transformation efficiency in this study was low due to rare random integration events.

Random integration of DNA into chromosomes by nonhomologous recombination has long been utilized for animal transgenesis. This is routinely used in mammalian systems [23]. In the insects, the distantly related sister group to *Daphnia*, some successes have been reported [24], [25], prompting us to apply this method for transgenesis of *Daphnia*. In the future, as reported in various organisms including insects [26], [27], [28], the utilization of transformation vectors with transposable elements may be useful to improve the transformation efficiency in *Daphnia*. Alternatively, vectors with recognition sites for endonuclease I-SceI that have been used for transgenesis in vertebrates [29] and even in an ancestral organism, sea anemone [30], may be helpful to enhance random integration of DNA in *Daphnia*. This study will enhance further development to improve the transgenesis rates. The ability to generate transgenic daphnids will be very important in facilitating utilization of emerging daphnid EST and genomic sequences. In turn, this will lead to a better understanding of the ecology, evolutionary biology, and molecular biology of daphnia.
